# Supplementary material for: Stacking Model‐Based Classifiers for Dealing With Multiple Sets of Noisy Labels
Source: Biom J. 2025 Mar 12;67(2):e70042. doi: 10.1002/bimj.70042 (PMC11898607; doi:10.1002/bimj.70042)
Supplement: Supplementary file 1 — Supporting Information [file BIMJ-67-e70042-s001.zip › density-based-ensemble-model-main/competitors_real_data/netlab-master/netlab-master/help/gtm.htm]

Netlab Reference Manual gtm


# gtm

## Purpose

Create a Generative Topographic Map.

## Synopsis

```
net = gtm(dimlatent, nlatent, dimdata, ncentres, rbfunc)
net = gtm(dimlatent, nlatent, dimdata, ncentres, rbfunc, prior)
```

## Description

`net = gtm(dimlatent, nlatent, dimdata, ncentres, rbfunc)`,
takes the dimension of the latent space `dimlatent`, the
number of data points sampled in the latent space `nlatent`, the
dimension of the data space `dimdata`, the number of centres in the
RBF model `ncentres`, the activation function for the RBF
`rbfunc`
and returns a data structure `net`. The parameters in the
RBF and GMM sub-models are set by calls to the corresponding creation routines
`rbf` and `gmm`.

The fields in `net` are

```
  type = 'gtm'
  nin = dimension of data space
  dimlatent = dimension of latent space
  rbfnet = RBF network data structure
  gmmnet = GMM data structure
  X = sample of latent points
```

`net = gtm(dimlatent, nlatent, dimdata, ncentres, rbfunc, prior)`,
sets a Gaussian zero mean prior on the
parameters of the RBF model. `prior` must be a scalar and represents
the inverse variance of the prior distribution. This gives rise to
a weight decay term in the error function.

## See Also

`gtmfwd`, `gtmpost`, `rbf`, `gmm`

---

**Pages:**
Index


---

Copyright (c) Ian T Nabney (1996-9)
